# Supplementary material for: Identification of photoperiod-induced specific miRNAs in the adrenal glands of Sunite sheep (Ovis aries)
Source: Front Vet Sci. 2022 Jul 22;9:888207. doi: 10.3389/fvets.2022.888207 (PMC9354845; doi:10.3389/fvets.2022.888207)
Supplement: Supplementary file 1 [file Data_Sheet_1.ZIP › Supplementary file/miRDeep2 file.zip!/miRDeep2 file/Know_Structure/SPLP42A3/oar-mir-19b.pdf]

miRBase precursor : oar-mir-19b
Total read count : 1477
oar-miR-19b read count : 1474
remaining reads : 3

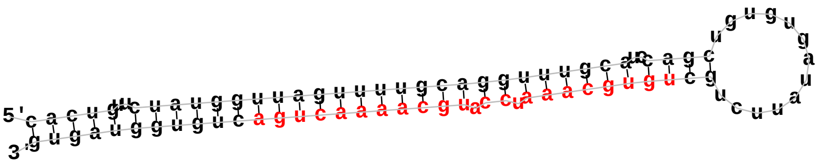

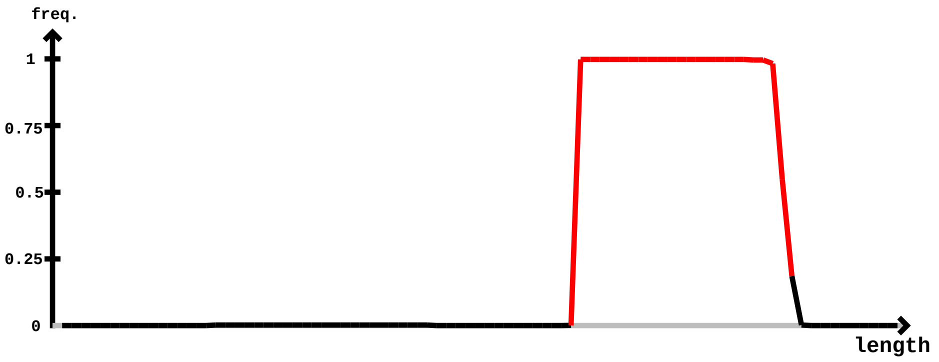

oar-miR-19b

```
5'- cacuguucuaugguuaguuuugcagguuugcauccagcugugugauauucugcugugcaaauccaugcaaaacugacugugguagug   -3'    exp
    (((((..(((((((((((((((((((((((((..((((.............)))))))))).)).))))))))))))))))))))))    reads      mm         sample
    ...............aguuuugcagguuugcauccagc.................................................    3          0          seq
    ....................................................cugugcaaauccaugcaaaacu.............    1          0          seq
    .....................................................ugugcaaauccaugcaaa................    3          0          seq
    .....................................................ugugcaaauccaugcaaaac..............    20         0          seq
    .....................................................ugugcaaauccaugcaaaacu.............    612        0          seq
    .....................................................ugugcaaaucUaugcaaaacu.............    29         1          seq
    .....................................................ugugcaaaucUaugcaaaacug............    57         1          seq
    .....................................................ugugcaaauccaugcaaaacug............    478        0          seq
    .....................................................ugugcaaauccaugcaaaacuga...........    216        0          seq
    .....................................................ugugcaaaucUaugcaaaacuga...........    55         1          seq
    .....................................................ugugcaaauccaugcaaaacugaU..........    3          1          seq
```
